# Molecular and Immunological Diagnostic Techniques of Medical Viruses

**DOI:** 10.1155/2020/8832728

**Published:** 2020-09-04

**Authors:** Daniel Hussien Reta, Tesfaye Sisay Tessema, Addis Simachew Ashenef, Adey Feleke Desta, Wajana Lako Labisso, Solomon Tebeje Gizaw, Solomon Mequanente Abay, Daniel Seifu Melka, Fisseha Alemu Reta

**Affiliations:** ^1^School of Veterinary Medicine, Wollo University, Dessie, Ethiopia; ^2^Institute of Biotechnology, Addis Ababa University, Addis Ababa, Ethiopia; ^3^Department of Microbial, Cellular and Molecular Biology, College of Natural and Computational Sciences, Addis Ababa University, Addis Ababa, Ethiopia; ^4^Department of Pathology, College of Health Sciences, Addis Ababa University, Addis Ababa, Ethiopia; ^5^Department of Medical Biochemistry, College of Health Sciences, Addis Ababa University, Addis Ababa, Ethiopia; ^6^Department of Pharmacology and Clinical Pharmacy, School of Pharmacy, College of Health Sciences, Addis Ababa University, Addis Ababa, Ethiopia; ^7^Department of Biology, College of Natural and Computational Sciences, Jigjiga University, Jigjiga, Ethiopia

## Abstract

Viral infections are causing serious problems in human population worldwide. The recent outbreak of coronavirus disease 2019 caused by SARS-CoV-2 is a perfect example how viral infection could pose a great threat to global public health and economic sectors. Therefore, the first step in combating viral pathogens is to get a timely and accurate diagnosis. Early and accurate detection of the viral presence in patient sample is crucial for appropriate treatment, control, and prevention of epidemics. Here, we summarize some of the molecular and immunological diagnostic approaches available for the detection of viral infections of humans. Molecular diagnostic techniques provide rapid viral detection in patient sample. They are also relatively inexpensive and highly sensitive and specific diagnostic methods. Immunological-based techniques have been extensively utilized for the detection and epidemiological studies of human viral infections. They can detect antiviral antibodies or viral antigens in clinical samples. There are several commercially available molecular and immunological diagnostic kits that facilitate the use of these methods in the majority of clinical laboratories worldwide. In developing countries including Ethiopia where most of viral infections are endemic, exposure to improved or new methods is highly limited as these methods are very costly to use and also require technical skills. Since researchers and clinicians in all corners of the globe are working hard, it is hoped that in the near future, they will develop good quality tests that can be accessible in low-income countries.

## 1. Introduction

Viruses are small segments of nucleic acid, deoxyribonucleic acid (DNA), or ribonucleic acid (RNA) within a protein coat or lipoprotein coat (envelope). Viruses require host resources for their replication because they are obligate intracellular parasites. Once viruses enter the host cells, they take over or hijack the cells' biosynthetic machineries for the replication of their genomes and other components [1, 2].

Viral infections are the most common cause of human diseases. Millions of people are still dying because of human immunodeficiency virus (HIV) and hepatitis viruses worldwide. The emerging viruses are also causing serious problems in human population. For example, avian influenza A (H5N1) in 1997, the severe acute respiratory syndrome-coronavirus (SARS-CoV) in 2002–2003, pandemic swine influenza A (H1N1) virus in 2009, Ebola virus in 2014, Zika virus (ZIKV) in 2015, and pandemic SARS-CoV-2 recently, among others, have caused several outbreaks in different countries [3–9].

The morbidity and mortality rates of human viral infections are significantly high [10]. For example, the pandemic swine influenza A (H1N1) infection in 2009 occurred in 214 countries with more than 18,036 deaths [5]. In 2010 alone, the number of human deaths due to rabies globally was estimated to be 61,000, with 84% of the deaths occurred in rural areas [11]. In 2013, approximately, 35,000,000 people were infected with HIV worldwide [10]. The World Health Organization (WHO) reported 1.34 million deaths of viral hepatitis in 2015 [12]. As on 6^th^ January 2015, H5N1 viruses have killed 402 out of 694 laboratory-confirmed human infections in 16 countries [13], with a mortality rate of around 58%. Recently, the world is challenged by the novel coronavirus disease 2019 (COVID-19). The disease is caused by the novel coronavirus (SARS-CoV-2). The pathogen first emerged in Wuhan city, Hubei province, China, which has now quickly gained worldwide spread [9, 14]. On 11^th^ March 2020, the WHO declared the COVID-19 outbreak a global pandemic. According to the WHO, 9, 129, 146 confirmed cases of COVID-19 have been reported worldwide, including 473, 797 deaths since 31^st^ December 2019 and as of 24^th^ June 2020 [15]. Therefore, good diagnostic techniques are required to detect these viral infections early and accurately. Early and accurate detection of viral diseases plays a significant role in selecting appropriate therapy timely, minimizing therapy costs, minimizing unnecessary loss of human lives, and controlling the disease. It also helps to develop appropriate disease prevention and treatment strategies, like development of antiviral vaccines and new therapeutic agents [14, 16, 17].

Traditionally, laboratory diagnoses of medical viruses are carried out by isolating viruses in embryonated chicken eggs, in tissue culture, or in laboratory animals and visual examination of viral particles in sample using electron microscopy among others [16]. Many conventional diagnostic tools tend to be cumbersome, time-consuming, expensive, and poorly reproducible [18, 19]. In contrast, molecular techniques have revolutionized diagnostic virology by detecting the presence or absence of viral nucleic acids in a patient's sample [18]. Immuno-based techniques still play a great role for the detection and serosurveillance of human viral infections despite the fact that many of the traditional methods are replaced by nucleic acid-based techniques [20]. Immunological methods detect viral infections by identifying antiviral antibodies or viral antigens in clinical samples [21, 22]. Here, we describe some of the molecular and immunological diagnostic approaches for the detection of medical viruses.

## 2. Molecular Diagnostic Techniques of Medical Viruses

Nucleic acid-based molecular detection techniques have revolutionized diagnostic virology with their faster, highly sensitive, and highly specific diagnosis [14, 23, 24]. Since these methods detect specific nucleic acid sequences, nucleic acid-based diagnostic tests can be applied for the detection of virtually any virus that affects humans [1].

### 2.1. Nucleic Acid-Based Amplification Techniques

Molecular techniques that involve the amplification of viral genomic material are extremely sensitive and specific, provide rapid diagnosis, and allow the detection of several viruses at same time [16]. Nucleic acid amplification techniques are very useful for the detection of viruses that are uncultivable or difficult and harmful to culture, slow growing viruses in culture, and viruses that display antigenic variations [1, 25]. The nucleic acid amplification tests are very popular in the diagnosis of viral infections caused by several viruses, including hepatitis C virus (HCV), human immunodeficiency virus (HIV), dengue virus, Epstein–Barr virus (EBV), influenza viruses, Zika virus (ZIKV), Ebola virus, and coronavirus [26–32]. Several nucleic acid amplification methods are currently available for the laboratory diagnosis of viral infections worldwide, and their advantages and limitations will be summarized in Table 1.

#### 2.1.1. Polymerase Chain Reaction (PCR)

PCR is a typical example of nucleic acid amplification assay. It has revolutionized the field of molecular diagnosis since developed by Mullis and Faloona [50]. PCR is based on extraction and purification of DNA molecule and exponential amplification of the target sequence, using a thermostable DNA polymerase and two specific oligonucleotide primers. After the PCR reaction, the amplified product can be detected by several techniques, including gel electrophoresis, colorimetric methods, and sequencing [10, 51, 52]. Since its inception, PCR has been used for the detection of human viral infections with overall clinical sensitivity ranging from 77.8% to 100% and clinical specificity ranging from 89% to 100% [28, 53–55]. These reports suggest that PCR can be employed for the detection of medical viruses in a variety of specimen types. Conventional PCR is still in use by some clinical laboratories worldwide, but it is rapidly replaced by more advanced variants of the technique.

PCR is a highly versatile technique. A number of variants of the conventional PCR have been developed, but the most important variants are reverse transcription-PCR and real-time PCR [1, 10]. The first method was devised to amplify ribonucleic acid (RNA) targets [1]; the second technique was introduced to quantify deoxyribonucleic acid (DNA) in real time throughout the PCR reactions [56].

#### 2.1.2. Reverse Transcription-PCR (RT-PCR)

RT-PCR was designed to amplify RNA targets. In this technique, reverse transcriptase (RT) is used to convert viral RNA targets into complementary DNA (cDNA), and then the resulting cDNA is amplified by conventional PCR. Since its development, RT-PCR has been used for the diagnosis of human infection by RNA viruses. Conventional RT-PCR demonstrated overall sensitivity ranging from 73% to 100% and specificity ranging from 99% to 100% in the detection of viral infection [29, 57, 58]. These data indicate that RT-PCR is an excellent technique for the diagnosis of human infection by RNA viruses. Nowadays, however, the method is not used commonly in clinical specimens owing to its high cost and time-consuming process [14].

#### 2.1.3. Real-Time PCR

**Figure 1:**
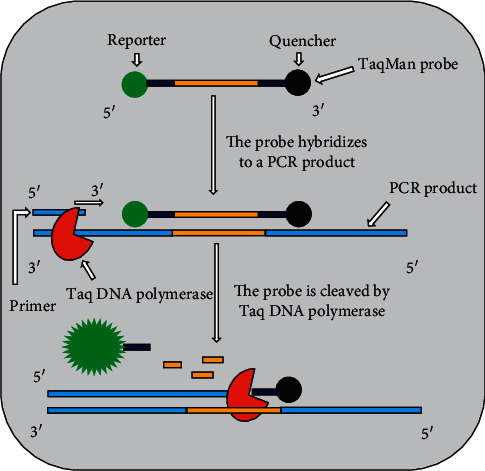
Schematic of the use of TaqMan probe in real-time PCR.

**Figure 2:**
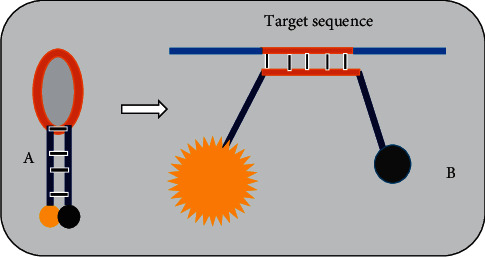
Diagram of molecular beacon. (A) The molecule forms hairpin when free in solution. This brings fluorophore (yellow ball) and quencher (black ball) in close proximity, so that no fluorescent light is detected. (B) The molecule hybridizes to the target sequence. This separates the fluorophore and quencher and leads to emission of fluorescent light.

In real-time PCR system, viral nucleic acid amplification and detection steps are carried out at the same time. The detection of the amplification product is relied on the amount of fluorescence emission from the specimen. The fluorescence emission from the specimen is monitored by special thermal cycler. The computer, with appropriate software connected to the thermal cycler, records the data and produces an amplification plot at every reaction cycle [51, 59]. The detection and quantification of amplification products can be done by using SYBR green, the TaqMan, and molecular beacon chemistries. The SYBR green dye binds to the minor groove of double-stranded DNA (dsDNA) product and upon excitation by appropriate light, it exhibits improved fluorescence, which is directly proportional to the accumulated dsDNA product. The TaqMan probe is a DNA oligonucleotide with a fluorescent dye termed reporter attached to one end (5′ base) and quencher on the other (3′ base) (Figure 1). TaqMan probes are designed to hybridize to an internal region of a PCR product. During the annealing stage of the PCR, both the primer and the TaqMan probe bind to the template strand. When the Taq DNA polymerase extends the primer, the polymerase cleaves the probe by its the 5′-3′ exonuclease activity. Cleavage of the probe leads to the release of the fluorescent dye (Figure 1), resulting in fluorescence emission. The amount of fluorescence is directly proportional to the PCR product. Molecular beacon is a small DNA molecule with a fluorescent dye at the 5′ end and a quencher at the 3′ end. The sequences at the very 3′ and 5′ ends are complementary to each other. The internal part of the molecule is designed to be complementary to the target sequence of a PCR product. When molecular beacon is free in solution, it will adopt a hairpin structure. This brings the fluorophore and quencher in close proximity, leading to absorption of emitted light of the florescent dye by the quencher and hence fluorescence is not detected (Figure 2 A). However, when a molecular beacon hybridizes to the target sequence, the fluorophore and quencher are separated, leading to the emission of fluorescence (Figure 2 B). The amount of fluorescence is directly proportional to the PCR product [16, 42, 51, 60].

Owing to high sensitivity and specificity, short turnaround time for results, and ease of performance [33, 61], most laboratories across the globe employ real-time PCR for the detection and quantification of medical DNA and RNA viruses in clinical specimens. For example, Boppana et al. [39] used real-time PCR for the detection of cytomegalovirus (CMV) in liquid saliva with overall sensitivity of 100% and specificity of 99.9%, compared with standard rapid culture. The method was also employed for the diagnosis of primary of EBV infection with overall sensitivity of 95.7% and specificity of 100%, compared to serologic assays [62]. Real-time PCR was also served to determine viral load in herpes simplex encephalitis patients [40]. The determination of viral loads in patient specimens is crucial as it provides prognostic and predictive information. In this study, patients with higher viral loads in their cerebrospinal fluid (CSF) found to require acyclovir therapy for a longer duration and had a poorer clinical outcome than the patients with lower viral loads in their CSF [40]. The assay can also be used for the multiplex identification of different viruses. Both TaqMan probe and molecular beacon play crucial roles for multiplex identification of different viruses in a single PCR reaction. In multiplexing assays, different probes/beacons are labeled with different fluorescent dyes [41]. In multiplex assay, sensitivity of 100% and specificity of 99.6% were reported, compared to immunofluorescence assay, for real-time PCR in the detection of human adenovirus B, C, and E in the throat swab samples [63]. Ramamurthy and his colleagues [33] compared multiplex real-time PCR with multiplex conventional PCR for the detection of neurotropic viruses (CMV, EBV, herpes simplex virus types 1 and 2 (HSV-1 and HSV-2) Japanese encephalitis virus (JEV), and varicella-zoster virus (VZV)) in CSF. Out of 147 CSF samples collected from patients with neurological disorders, real-time PCR detected viral pathogens in 88 samples while conventional PCR could only detect the viruses in six samples, suggesting that real-time PCR has higher sensitivity than conventional PCR. Qiu et al. [64] developed a triplex quantitative real-time PCR assay for rapid and differential detection of human adenovirus (hAdV) serotypes 2, 3, and 7 for potential clinical use. The analytical sensitivity (limit of detection; LoD) of this assay was 10^2^ DNA copies/reaction for each of serotypes and no cross-reactions with other respiratory pathogens. The authors concluded that the assay is sensitive and specific and has the potential for clinical use in the rapid and differential detection and quantitation of hAdV serotypes 2, 3, and 7 in human specimens.

By the incorporation of reverse transcription step, real-time PCR can be combined with the conventional reverse transcription PCR (RT-PCR) to form reverse transcription quantitative real-time PCR (RT-qPCR). RT-qPCR has a number of advantages over the conventional RT-PCR technique, including reduction of contamination, possibility of quantifying the amplicons, and quick assay time since there are no post-PCR processing activities [14, 51]. As a result, RT-qPCR is widely deployed for the detection and quantification of several RNA viruses in clinical specimens, including ZIKV, Ebola virus, coronavirus, HCV, respiratory syncytial virus (RSV), dengue virus, HIV-1, and influenza A virus [30–32, 65–69]. Recently, Corman et al. [32] developed RT-qPCR for the detection of SARS-CoV-2. The assay targeted envelope protein (E) gene and RNA-dependent RNA polymerase (RdRp) gene of SARS-CoV-2. High LoDs of 5.2 copies/reaction for E-gene and 3.8 copies/reaction for RdRp gene were demonstrated and no cross-reaction with other coronaviruses, suggesting the usefulness of the method for sensitive and specific diagnosis of COVID-19. The RT-qPCR assay (Quanty ZEBOV FAST assay) was evaluated for the detection of Ebola virus in clinical samples. CLONIT Srl (Italy) developed the assay during outbreak of Ebola in Sierra Leone, and it had overall sensitivity of 100% and specificity of 98.63%, compared to RealStar® Filovirus Screen RT-qPCR Kit 1.0 (Altona Diagnostics) [31]. Gueudin et al. [68] used RT-qPCR for diagnosis and monitoring of HIV-1 group O infection with LoD of 40 copies/ml and specificity of 100%. The method was applied for ZIKV detection in human serum and urine, and it had LoDs of 2.5 PFU/ml and 250 PFU/ml in urine and serum, respectively [30]. Júnior et al. [70] used RT-qPCR for the detection of respiratory viruses in outpatients with acute respiratory infection. They also compared the performance of RT-qPCR with indirect immunofluorescence assay (IFA). Accordingly, RT-qPCR managed to detect viral pathogens in 88 (88/162) nasopharyngeal aspirates, but IFA detected viral pathogens in only 33 (33/162) specimens. The data indicated that the use of RT-qPCR increased the viral detection by 33.9%. Today, several real-time RT-PCR kits are available commercially. For example, real-time-qPCR test developed by Cepheid AB (Sunnyvale, CA, USA) is commercially available for the qualitative detection of Ebola virus in EDTA venous whole blood or buccal swabs. The assay targets viral nucleoprotein and glycoprotein genes of Ebola Zaire virus. The assay has a LoD of 82 RNA copies/reaction with turnaround time of 98 minutes. Simplexa™ Dengue RT-PCR assay developed by Focus Diagnostics (Cypress, CA, USA) is a commercial kit for detection and typing of dengue virus serotypes 1, 2, 3, and 4 in human serum. The assay targets four serotype specific regions, namely, dengue 1 (nonstructural (NS)5 gene ), dengue 2 (NS3 gene), dengue 3 (NS5 gene), and dengue 4 (capsid gene). The LoDs of the assay are 0.16 PFU/ml, 2.0 PFU/ml, 0.2 PFU/ml, and 0.2 PFU/ml for dengue 1, dengue 2, dengue 3, and dengue 4, respectively. Real-Star Zika Virus RT-PCR kit 1.0 is available, developed by Altona Diagnostics (Hamburg, Germany), for qualitative detection of ZIKV specific RNA in human serum or urine. The LoD of the assay is 0.61 RNA copies/*μ*l. Abbott RealTime HCV quantitative assay developed by Abbott Laboratories (Rungis, France) is commercially available for HCV RNA quantitation in human serum and plasma. The target sequence for the assay is in the highly conserved 5′untranslated region (UTR) of the HCV genome. The LoD of the assay is 12 IU/ml when testing human plasma or serum. COBAS TaqMan HIV-1 test developed by Roche Diagnostics (Branchburg, USA) is commercially available for the quantitation of HIV-1 in human plasma. The real-time RT-PCR targets two highly conserved regions of the HIV-1 genome, namely, gag and long terminal repeat (LTR). The assay has LoD of 20 HIV-1 RNA copies/ml. Recently, several developers of diagnostic tests have developed real-time RT-PCR kits for COVID-19, and they are now seeking marking and emergency use authorization (EUA) from regulatory agencies. For example, Co-Diagnostics (Salt Lake City, USA) has developed real-time RT-PCR kit (Logix Smart COVID-19 test) for qualitative detection of nucleic acid from the SARS-CoV-2 in lower respiratory samples (e.g., bronchoalveolar lavage, sputum, and tracheal aspirate) and upper respiratory specimens (e.g., oropharyngeal swabs, nasal swabs, and nasopharyngeal swabs). The kit has received EUA from United States Food and Drug Administration (US FDA) and CE-IVD marking approval. The assay targets RdRp gene of SARS-CoV-2. The LoD of the assay is 9.35 × 10^3^ RNA copies/ml with thermocycler run time of 63–90 minutes, depending on PCR equipment. US Centers for Disease Control and Prevention (CDC) has developed three real-time RT-PCR assays for the detection of SARS-CoV-2 genetic material in upper and lower respiratory specimens, and this panel has been granted EUA by the US FDA. CDC real-time RT-PCR panel targets 3 candidate regions of nucleocapsid (N) gene of SARS-CoV-2. The LoD of all assays are 5 RNA copies/reaction. The Agency for Science, Technology and Research (A∗STAR) and Tan Tock Seng Hospital (TTSH) (Singapore) have developed real-time RT-PCR test (Fortitude Kit 2.0) for qualitative detection of SARS-CoV-2 genetic material in oropharyngeal swabs. The kit has received Singapore Health Sciences Authority's (HAS) provisional authorization for clinical use. The US FDA has not yet approved the kit for clinical use. The assay has LoD of 1000 RNA copies/ml in in oropharyngeal swabs. The kit developers have not yet disclosed the target gene for this assay. BGI Group (Beijing, China) has developed real-time fluorescent RT-PCR kit for the qualitative detection of nucleic acid from the SARS-CoV-2 in throat (oropharyngeal) swabs, nasal swabs, nasopharyngeal swabs, and other respiratory specimens. The company has received an EUA form the China's National Medical Product Administration and the US FDA for its test kit. The assay targets open reading frame 1a and b (ORF1ab) genes of SARS-CoV-2. It has LoD of 150 copies/ml in throat samples with turnaround time of 4 hours. RADI COVID-19 Real-Time PCR kit developed by KH Medical (Korea) has CE-IVD marking and is used for qualitative detection of SARS-CoV-2 in human nasal swab or sputum sample. The assay targets two genes, namely, spike protein (S) gene and RdRP gene of SARS-CoV-2. It has LoD of 0.66 copies/*μ*l with turnaround time of 80 minutes.

#### 2.1.4. Transcription-Based Amplification Methods

Transcription-based amplification method includes nucleic acid sequence-based amplification (NASBA) and transcription-mediated amplification (TMA). NASBA and TMA are similar to each other. They are isothermal amplification methods. The entire amplification process is carried out at the temperature of 41°C. In both cases, the viral RNA target is first converted into cDNA with RT and then RNA polymerase synthesizes multiple copies of viral RNA product. The only difference between TMA and NASBA in the amplification process is two enzymes (RT and RNA polymerase) are utilized in case of TMA while NASBA utilizes three enzymes (avian myeloblastosis virus reverse transcriptase (AMV-RT), RNase H, and T7 RNA polymerase) [42, 51].

**Figure 3:**
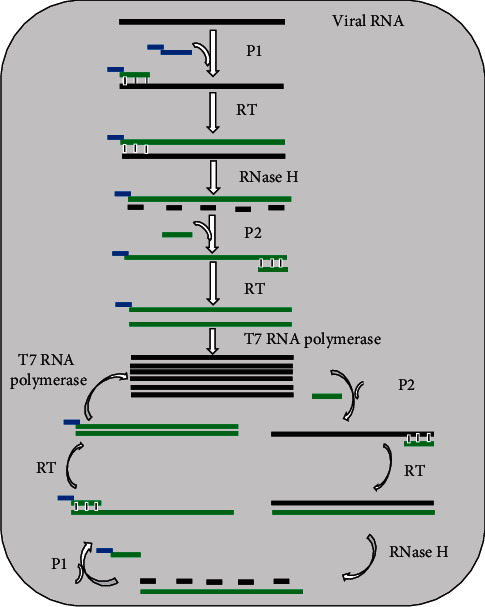
Schematic of the principle of NASBA. Abbreviations: P1, primer 1; P2, primer 2; RT, reverse transcriptase.

As depicted in Figure 3, in the NASBA process, three enzymes and two primers work together to exponentially amplify a target viral RNA. Primer 1 (P1) carries at its 5′ end T7 RNA polymerase promotor region and at its 3′ end, P1 carries sequence that is complementary to a target viral RNA sequence. Primer 2 (P2) carries a sequence complementary to cDNA strand. The amplification reaction begins with the production of cDNA copy of the viral RNA by RT using P1. RNase H degrades the viral RNA from RNA-DNA hybrid molecules. Then, RT synthesizes dsDNA molecules using P2 and the released DNA strand. Finally, T7 RNA polymerase uses dsDNA molecules as templates to transcribe many viral RNA copies. The above cycle is repeated several times, resulting in the accumulation of many viral RNA copies and ds DNA molecules. The amplified product can either be detected by gel electrophoresis at the end of the assay or in real time using molecular beacon [16, 42, 43, 71]. Transcription-based amplification methods have several advantages, for example, they do not require a thermal cycler, so developing countries and budget-restricted laboratories can afford to perform the assays, they have rapid kinetics (requires fewer cycles), and they produce a single-stranded RNA product that is suitable for detection by various techniques [42, 51, 71, 72]. Transcription-based amplification methods are suitable for the diagnosis of human viral infections caused by RNA viruses. They can amplify viral genomic RNA, messenger RNA, or ribosomal RNA [51, 71, 73]. Ayele et al. [44] developed NASBA assay that uses gag-based molecular beacons to distinguish between HIV-1 subtype C (C and C′) circulating in Ethiopia. The assay demonstrated high levels of sensitivity and specificity for both beacons (90.5% sensitivity and 100% specificity for the C beacon and 100% sensitivity and 95.2% specificity for the C′ beacon) by considering sequencing as gold standard for genotyping. Moore et al. [74] also used NSABA for the detection of influenza A H5N1 virus in clinical specimens with a LoD of 10 RNA copies/*μ*l along with the same sensitivity as RT-PCR and average turnaround time of 4 hours. The NASBA assay was also used for the detection of dengue viral RNA with LoD of 1 PFU/ml for all of 4 dengue virus serotypes, no cross-reaction with JEV, and turnaround time of 3 hours [27]. Ender et al. [26] used TMA for screening of blood donations for HIV-1 and HCV RNA. The TMA assay had LoDs of 16.2 IU/ml for HIV-1 and 3.5 IU/ml for HCV. A multiplex NASBA assay was used for simultaneous detection of HIV-1 and HCV in plasma samples. The LoD of the assay for both HIV-1 and HCV was determined to be 1000 copies/ml and no cross-reactions with other selected viruses [45]. Swenson and his colleagues [75] used real-time TMA for the detection of HSV-1 and HSV-2 in lesion swab specimens with overall sensitivities of 98.2% and 99.4%, respectively, and specificity of 97.8% and 94.5%, respectively, compared to culture. In one study, real-time NASBA assay was more sensitive than the conventional RT-PCR in the detection of norovirus. In this study, RT-PCR detected 10 pg of standard viral RNA, while the real-time NASBA assay could detect 100 fg of standard viral RNA [76]. These data indicate that the assays are sensitive, specific, and cost-effective for the detection of human infection by RNA viruses.

TMA-based assays for the detection of HCV and HIV-1 are commercially available, developed by Hologic (San Diego, CA, USA). The Aptima HCV RNA qualitative assay is used for the detection of HCV RNA in human plasma or serum. The assay utilizes TMA to amplify conserved regions within the 5′-UTR of the HCV genome. The assay has LoD of 7.5 IU/ml with a specificity of 99.6%. NASBA-based kits for detection of HIV-1, CMV, enterovirus, and RSV are also commercially available, developed by bioMérieux Clinical Diagnostics. The NucliSens Easy Q RSV A and B assay is developed by bioMérieux (Marcy l'Etoile, France), and it is used for qualitative detection of RSV in respiratory samples of different types. The assay is based on real-time NASBA, and it targets F gene of RSV. Moore et al. [77] evaluated the performance of the commercial test kit using 508 respiratory specimens that were tested by direct immunofluorescence and culture. The assay was found to be more sensitive than culture and immunofluorescence assay. The sensitivity and specificity of the assay were determined to be 99% and 87%, respectively, compared to immunofluorescence assay with turnaround time of <4 hours.

#### 2.1.5. Loop-Mediated Isothermal Amplification (LAMP)

LAMP is another isothermal nucleic acid amplification method that is extensively utilized for sensitive, specific, rapid, and cost-effective detection of both DNA and RNA viruses in human specimens. The method was first developed by Notomi et al. [78] and rapidly gained popularity in diagnostic virology. The method employs four to six unique primers and DNA polymerase with strand-displacement activity to amplify target DNA [78, 79]. The addition of RT in LAMP reaction (RT-LAMP) permits the amplification of RNA target [80]. Primer sets for LAMP initially reported by Notomi et al. [78] include the forward inner primer (FIP), backward inner primer (BIP), forward outer primer (F3), and backward outer primer (B3). The primers are specifically designed to recognize six precise regions from a targeted nucleic acid sequence. Nagamine et al. [79] later added two loop primers, namely, forward loop primer (LF) and backward loop primer (LB), to accelerate LAMP assay. Owing to the use of four to six specific primers, LAMP assay has outstanding sensitivity and specificity in the detection of target nucleic acids [79, 81]. A detailed description of the LAMP reaction mechanism is available in reviews by Becherer et al. [81], Tomita et al. [82], and Silva et al. [83], which use illustrations to explain the mechanism. The LAMP reaction is performed in constant temperature between 60–65°C, alleviating the need for expensive specialized equipment. The method requires only inexpensive heating block or water bath, making it very useful under poor laboratory settings [84]. The LAMP reaction takes turnaround time of <1 hour and the amplified product can be detected by several methods, including the real-time measurement of the turbidity caused by precipitated magnesium pyrophosphate using a turbidimeter, visual detection of magnesium pyrophosphate precipitation following completion of the reaction, detection of fluorescence under ultraviolet light or natural light by adding an intercalating fluorescent dye to the final reaction mixture, and visualization of the bands with various sizes using agarose gel electrophoresis [84–87].

LAMP assay has been successfully utilized to the rapid detection of a number of DNA viruses in human specimens, such as HSV-1 with LoD of 10 copies of HSV-1 DNA/*μ*l and no cross-reactions with other selected viruses [85], hAdV40 and hAdV41 with LoD of between 50 and 100 copies of DNA/reaction, no cross-reactions with other selected viruses, and turnaround time of 60 minutes [88], EBV with sensitivity of 86.4%, specificity of 100%, compared to serological assay, and only 45 minutes of amplification of the target sequences [89], and CMV with LoD of 10 DNA copies/*μ*l, no cross-reactivity with other viruses, and turnaround time of 1 hour after RNA extraction [90].

The utility of LAMP is expanded by merging it with reverse transcription (RT) into RT-LAMP to allow the rapid detection of RNA viruses in clinical specimens. Recently, for instance, Huang et al. [91] developed a rapid RT-LAMP assay for diagnosis of SARS-CoV-2 with LoD of 80 copies of viral RNA/ml in a sample within a 30 minutes reaction. This assay was validated by using 16 clinical samples (8 positives and 8 negatives) that were also tested by RT-qPCR. The testing results of the assay were consistent with RT-qPCR method, suggesting RT-LAMP assay can be used for rapid, simple, cost-effective, and sensitive detection of SARS-CoV-2 in respiratory samples. Similarly, Lu et al. [92] developed the RT-LAMP method for rapid detection of SARS-CoV-2 with LoD of 30 RNA copies/reaction and turnaround time of 40 minutes . Further, Baek et al. [93] developed a rapid RT-LAMP assay for early detection of SARS-CoV-2. The assay has LoD of 100 RNA copies/reaction, which is close to that of RT-qPCR with a rapid detection span of 30 minutes . RT-LAMP assay has also been developed to detect Middle East respiratory syndrome coronavirus (MERS-CoV) with LoD of 3.4 copies of MERS-CoV RNA/reaction along with the same sensitivity as MERS-CoV RT-qPCR, no cross-reaction to other respiratory viruses, and results available in <1 hour [87]. Kurosaki et al. [94] detected acute Ebola virus infection by RT-LAMP coupled with a portable device. The sensitivity and specificity of the assay was 100% each, compared to RT-qPCR and turnaround time of 35 minutes . In one study, RT-LAMP was more sensitive than conventional RT-PCR and NASBA [95]. The assay has been also developed for rapid detection of dengue virus [84], influenza A (H1N1) pdm09 virus [96], H5N1 avian influenza virus [97], HCV [98], HIV-1 [99], RSV [46], and ZIKV [100] in clinical samples. RT-LAMP-based commercial test kits are available for the detection of SARS-CoV-2 in respiratory specimens. The assay is developed by Color Genomics (USA), and it uses three SARS-CoV-2 specific primer sets targeting N gene , E gene , and ORF1a region, respectively, and a fourth control primer set targeting human ribonuclease P (RNaseP). It has LoD of 0.75 copies of viral RNA/*μ*l with 70 minutes reaction. The assay received EUA from the US FDA in respiratory specimens. Abbott Diagnostic Scarborough, Inc. (USA) has also developed RT-LAMP-based test (ID NOW™ COVID-19 assay) for direct detection of SARS-CoV-2 in nasal, nasopharyngeal, or throat swabs. The kit has received EUA from US FDA. The assay targets RdRp gene of SARS-CoV-2. The LoD of the test is 125 genome equivalents (GE)/ml with positive results in <5 minutes and negative results in 13 minutes . LAMP primer sets such as the Loopamp primer set for avian flu H5 and H7 and FluA influenza are commercially available from Eiken Chemical Co., Ltd. (Japan).

### 2.2. DNA Microarrays

DNA microarray technologies have the capacity to identify medical viruses [101]. In DNA microarray diagnosis, fluorescently labeled viral nucleic acids in a test sample are used to screen an array of oligonucleotide probes immobilized on a solid surface (e.g., glass slide). The oligonucleotide probes used here are specific for the genome of the target virus. The results of hybridization between immobilized probes and target sequences labeled with fluorescent dyes are detected and quantified by fluorescence-based detection [16, 51, 72].

Extensive literature exists demonstrating the utility of DNA microarray for the detection of medical viruses in human specimens. Chiu et al. [102] used DNA microarray for high-throughput multiplex detection of viruses in nasopharyngeal aspirate samples originated from children infected with respiratory viruses. The assay demonstrated overall sensitivity of 87% to 90% and specificity of ≥99% in the detection of RSV, influenza A virus, and rhinovirus/enterovirus compared to RT-PCR. In one study, DNA microarray was utilized for simultaneous detection of HSV-1/2, VZV, EBV, CMV, human herpesvirus-6 types A and B (HHV-6 A/B), and adenovirus in clinical samples with LoD of 10 GE/reaction for each virus without cross-reactivity [103]. DNA microarray was also used to identify viral causes of meningitis and encephalitis with overall sensitivity of 93% and specificity of 100%, compared to single-virus PCR [104]. DNA microarray was also utilized for high-throughput multiplex detection of gastrointestinal viruses [105], viruses transmitted by small mammals and arthropods [106], herpesviruses, enteroviruses and flaviviruses [107], HIV-1, HIV-2, and hepatitis viruses [108] and dual infection with two dengue virus serotypes [109] in human specimens. DNA microarray was used to identify and genotype drug-resistant mutations of HIV [110, 111] to detect and genotype drug-resistant hepatitis B virus (HBV) mutations [112], to detect and genotype SARS coronavirus [113], and to detect and determine lineage of influenza B viruses [114]. During an outbreak of SARS in China in 2002, DNA microarray also served for the discovery of a new member of the coronavirus family [115].

DNA microarray technology is a high-throughput tool as it allows multiplex detection of a large number of potential viral pathogens in clinical specimens [102–105]. The technique does have a number of limitations nevertheless, including being too expensive to be used for routine clinical diagnosis, labor-intensive, and time-consuming (the hybridization process may take hours to days to complete). Nonspecific hybridization between test materials and immobilized probes can affect the sensitivity of the assay. In addition, designing of specific probes requires almost complete information of the genetic makeup the virus of interest. The assay detects only those viral pathogens that have target probes on the array [72, 116, 117].

### 2.3. Next-Generation Sequencing (NGS)

NGS finds itself very useful in diagnostic virology as it can directly analyze viral nucleic acid fragments extracted from clinical specimens [118, 119]. Generally, NGS involves preparation of test sample, sequencing of the target nucleic acid fragments using one of the available NGS platforms, and analysis of the sequence data using suitable bioinformatic tools [10, 120]. Several companies produce different NGS machines that use different methods of sequencing, reagents, and data analysis tools [119]. For example, pyrosequencing (Roche 454) detects release of pyrophosphate following incorporation of nucleotides in a DNA polymerization process. Illumina's NGS platforms detect release of fluorescent labels from incorporated nucleotides in a DNA polymerization process. The emerging technologies like Oxford nanopore (MinIon) platform sequences the target nucleic acid by sensing the ionic current of DNA/RNA molecules that pass through the nanopores [10, 119, 120]. Despite its high sequence error rate (up to 38.2%) [121], MinION nanopore sequencer has merits over other NGS platforms. Firstly, it can generate longer read lengths (up to 882 kb) in real time [122], making it suitable for whole genome sequencing of viral pathogens with short turnaround time [123, 124]. Secondly, it is portable and no Internet is required for analysis, making it deployable in the field during outbreaks of viral infections [125]. Thirdly, it has low capital cost, making it affordable in low-income countries and budget-restricted laboratories across the globe [121].

NGS has been used in diagnostic virology for several applications. Recently, Kustin et al. [126] used NGS for rapid and robust identification of respiratory viruses in clinical samples. It was applied to track influenza A (H1N1) pdm09 virus [127]. Dessilly et al. [128] used NGS for the detection of HIV-1 drug resistance mutations. NGS was also conducted to discover a new Ebola virus [129].

Unlike PCR and DNA microarray methods, NGS does not require prior knowledge of genomic sequences of the viral pathogens. It does not also require target specific PCR primers and oligonucleotide probes [126, 130]. However, the use of NGS in clinical laboratories is limited because of the following reasons: the turnaround time, the number of samples per run, cost of sequencers, and requirement of skills in bioinformatics [10, 128, 131].

## 3. Immunological Diagnostic Techniques of Medical Viruses

The humoral branch of the immune system makes antibodies in response to viral infections. This natural response of the human body against viral infection is utilized for the development of immunological diagnostic methods. Several immunological diagnostic techniques are available for the detection human viral infections in clinical samples, including enzyme-linked immunosorbent assay, western blotting, immunofluorescence assay, and hemagglutination inhibition assay. The principles of these assays rely on the formation of antigen-antibody complex and consist of clinical specimens, whole virus or viral antigen, and an indicator [10, 19, 20].

### 3.1. Enzyme-Linked Immunosorbent Assay (ELISA)

**Figure 4:**
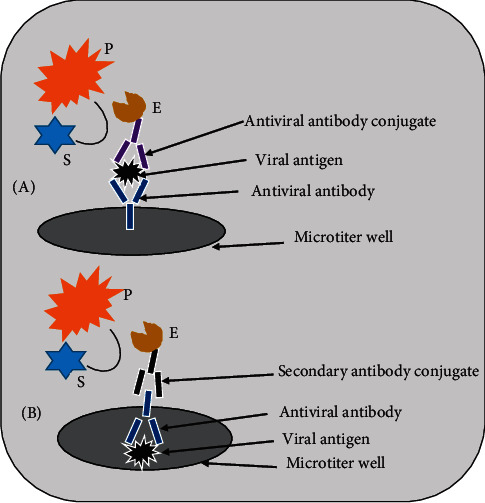
Diagram of the two principles of ELISA. (A) Sandwich ELISA. (B) Indirect ELISA. Abbreviations: E, enzyme; S, substrate; P, product.

In ELISA, enzyme conjugated antibody is utilized to detect the presence of specific antiviral antibody or viral antigen in human specimens. In positive sample, the reaction between an enzyme conjugated with an antibody and colorless chromogenic substrate leads to the formation of a colorful product. In the absence of antigen/antibody in the clinical specimen, no color is produced. The intensity of the color is directly proportional to the amount of antigen-antibody complex formed. The color change can be observed by the naked eye or read by a spectrophotometer, which can measure the absorbance. Several enzymes, including alkaline phosphatase, horseradish peroxidase (HRP), and *β*-galactosidase, have been used for ELISA. There are several variants of ELISA, but the two main types are antigen-capture ELISA (also called sandwich ELISA) and antibody-capture ELISA (also called indirect ELISA) [19, 132]. As illustrated in Figure 4, the first method detects viral antigen by immobilizing antibody specific for the viral protein of interest on a microtiter well [22]; the second technique detects antiviral antibody in a patient sample by coating whole virus or viral protein on a microtiter well [133].

ELISA is sensitive and specific, easy to perform, and has a short turnaround time for results. Consequently, the assay has been developed and extensively utilized for the detection and serosurveillance of human viral pathogens. Recently, Adams et al. [134] developed antibody-capture ELISA for the detection of SARS-CoV-2 IgM or IgG in human plasma samples. The assay was tested by using 40 plasma samples from RT-PCR-confirmed SARS-CoV-2 infected patients and 50 plasma samples from healthy control. It demonstrated overall sensitivity of 85%, compared to RT-PCR and specificity of 100% in the detection of anti-SARS-CoV-2 IgG or IgM in plasma samples. This assay detected IgG levels in all of RT-PCR positive individuals from ≥10 days following symptoms onset with a sensitivity of 100%. Similarly, Colavita et al. [135] developed antibody-capture ELISA for the detection of anti-SARS-CoV-2 IgG, IgM, and IgA in serum samples. The assay was validated using 553 serum samples collected from suspected and confirmed SARS-CoV-2 infection cases, healthy donors, and patients positive for other infections or autoimmune conditions. The assay showed overall sensitivity of 91.7% and 97.9% for the detection of IgG and IgA in serum sample, respectively, and specificity of >96% for all antibody types, compared to IFA reference test. Chen et al. [22] also developed antigen-capture ELISA for the detection of MERS-CoV in clinical specimens. The assay demonstrated a LoD of <1 ng of MERS-CoV-recombinant nucleocapsid protein/ml and specificity of 100%. Antigen-capture ELISA was developed for rapid detection of dengue virus NS1 and differentiation of DENV serotypes in human specimens. The overall sensitivity and specificity of the test were 84.85% and 100%, respectively, compared to RT-qPCR, and the sensitivity rates for serotyping were 88.2%, 94.7%, 75%, and 66.6% for DENV serotype 1 (DENV1), DENV2, DENV3, and DENV4, respectively, with no cross-reactivity among serotypes [136]. ELISA was also employed for the detection of several other medical viruses, including Ebola virus [133], HSV-2 [137], SARS-CoV [138], hepatitis viruses [139], H5N1 influenza virus [140], and ZIKV [141].

Commercial antibody-capture ELISA-based test kit (Anti-ZIKV IgA, IgG or IgM ELISA) is available, developed by Euroimmun AG (Germany), for serodiagnosis of acute and past ZIKV infections. The assay uses ZIKV-specific NS1 recombinant antigen. The overall sensitivity and specificity of the assay are 100% and 94%, respectively, compared to RT-PCR. Creative Diagnostics (USA) also developed sandwich ELISA-based commercial kit (HIV 1 and 2 Ag/Ab ELISA kit) for qualitative determination of antigens or antibodies to HIV-1 and HIV-2 in human serum or plasma samples. The assay uses recombinant HIV antigens (HIV-1 glycoprotein (gp)41, gp120, and HIV-2 gp36) and anti-HIV viral gag protein p24 antibodies. The LoD of the assay for the detection of HIV p24 antigen is about 1pg/ml. Moreover, Bio-Rad (France) developed NS1 Ag capture ELISA-based commercial kit (Platelia Dengue NS1 Ag) for the qualitative or semiquantitative detection of dengue virus NS1 antigen in human serum or plasma samples. The assay employs anti-NS1 monoclonal antibody (Mab) as capture antibody and anti-NS1 Mab-HRP conjugate as detection antibody. The sensitivity rates of the assay related to virus serotype are 88.9%, 87.1%, 100%, and 93.3% for DENV1, DENV2, DENV3, and DENV4, respectively, compared to RT-PCR, and specificity of the assay is 100% for all serotypes. Recently, Euroimmun AG (Germany) has developed antibody-capture ELISA-based kit (Anti-SARS-Cov-2 ELISA IgG) for qualitative detection of IgG to SARS-CoV-2 in human serum or plasma samples. The assay uses recombinant S1 protein of SARS-CoV-2 as capture antigen. The assay has received EUA from US FDA for use in authorized laboratories. The estimated sensitivity and specificity of the assay are 90% and 100%, respectively, compared to nucleic acid amplification test. Epitope Diagnostics, Inc. (USA) has also developed two types of ELISA kits (COVID-19 IgG ELISA and COVID-19 IgM ELISA Kits) for the detection of anti-SARS-CoV-2 IgG and IgM in human serum samples, respectively. COVID-19 IgG ELISA kit uses SARS-CoV-2 recombinant antigen and HRP labeled anti-human IgG antibody. COVID-19 IgM ELISA employs anti-human IgM antibody and HRP labeled SARS-CoV-2 recombinant antigen. The assays have a LoD of 5IU/ml. The kits are approved by FDA for clinical and research use.

### 3.2. Western Blotting Analysis

**Figure 5:**
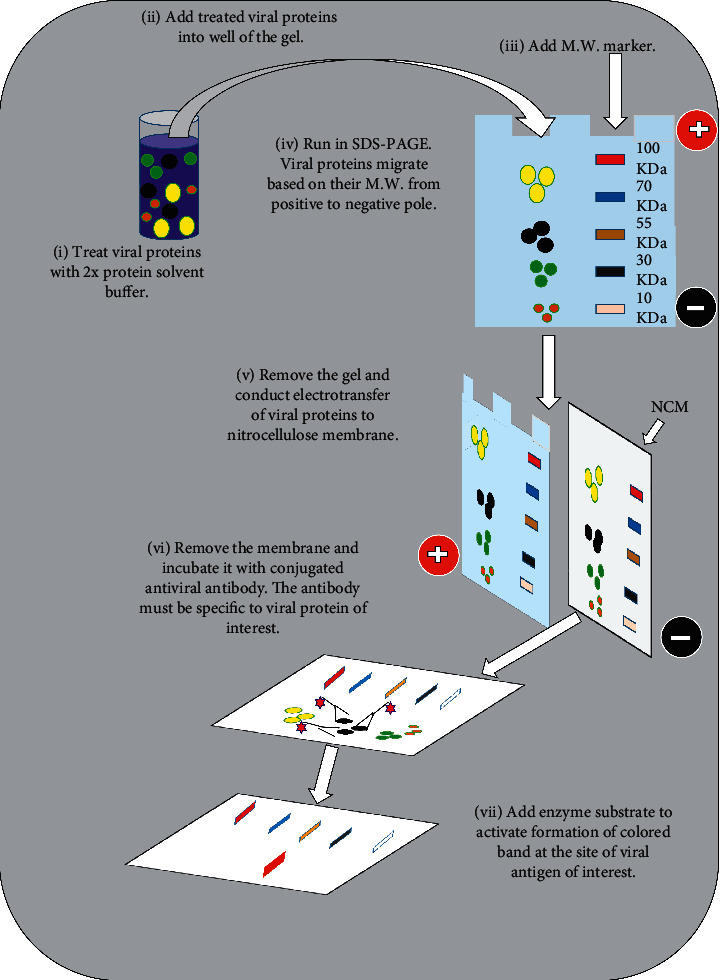
Schematic of immunoblot analysis of viral protein. Abbreviations: M.W., molecular weight; SDS-PAGE, sodium dodecyl sulfate-polyacrylamide gel electrophoresis; NCM, nitrocellulose membrane.

Western blotting (also known as immunoblotting) assay detects viral proteins or antiviral antibodies. For detection of viral proteins, denatured whole viral proteins are first separated by sodium dodecyl sulfate-polyacrylamide gel electrophoresis (SDS-PAGE). Viral proteins are then electrotransferred onto nitrocellulose membrane. The membrane is then incubated with enzyme conjugated antibodies specific for the viral proteins. If the viral proteins are bound by enzyme labeled antibody, addition of a chromogenic substrate leads to the formation of colored bands at the sites of the viral antigens (Figure 5) [19, 132]. For detection of antiviral antibodies, viral specific denatured proteins are electrophoretically blotted onto nitrocellulose membrane after subjected to SDS-PAGE. The membrane is then incubated with patient serum. If the patient serum contains antibodies against the viral proteins, they will bind to their specific viral proteins. The addition of enzyme conjugated secondary anti-human antibody and a chromogenic substrate results in the production of colored bands at the locations of the viral proteins [142].

Immunoblotting has been used in clinical diagnosis for serosurveillance and as confirmatory tests for human viral infection. He et al. [143] developed western blot assay for detection of antibodies against SARS-CoV in human serum samples. The assay demonstrated a sensitivity of 98.3% and specificity of 90.9%, compared to IFA. Western blotting assay was also used for the detection of anti-Chikungunya virus antibody in human serum. Sensitivity of 83.3% and specificity of 96.7% were demonstrated by the assay using 30 sera from confirmed Chikungunya virus infected patient and 30 normal sera [144]. In one study, western blotting was a promising method for surveillance of HIV-1 infection in resource-limited regions [145]. The assay was also used for the detection and confirmation of HCV and HIV infections [146–148]. Western blotting assay is also commercially available. For example, J. Mitra and Co. Pvt. Ltd (Mumbai, India) developed commercial kit (HIV 1 and HIV 2 western blot) for the detection of antibodies to HIV-1 and HIV-2 in human serum or plasma samples. The assay uses preblotted nitrocellulose membrane strips with resolved HIV-1 viral lysate and HIV-2 antigen (gp36). The assay has 100% sensitivity and 100% specificity when compared with licensed western blot test. GS HIV-1 Western Blot kit for the detection of antibodies to HIV-1 in human serum, plasma, or dried blood spots is also available developed by Bio-Rad Laboratories (Redmond, USA). The assay uses preblotted nitrocellulose membrane strips with resolved HIV-1 viral proteins. The assay has 100% sensitivity and 87.2% specificity, compared to licensed HIV-1 western blot test.

### 3.3. Immunofluorescence Assay

**Figure 6:**
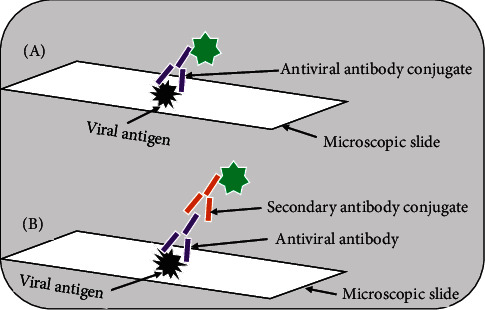
Schematic of the principles of immunofluorescence assays: (A) direct immunofluorescence assay; (B) indirect immunofluorescence assay.

Immunofluorescence assay is commonly conducted for the detection of viral antigens or antiviral antibodies in clinical samples. The assay is conducted in two formats: direct immunofluorescence assay (DFA) that detects viral antigens in patient sample [149] and indirect immunofluorescence assay (IFA) that detects antiviral antibody [150] or viral antigen [151] in clinical specimen. In the DFA, antibody that recognizes viral antigen is directly conjugated to fluorescent dye. In the IFA, viral antigen specific antibody is unlabeled and is detected with a second fluorescently labeled anti-human antibody (Figure 6). IFA is more sensitive than DFA because several fluorescently labeled anti-immunoglobulin antibodies bind to each antiviral antibody, increasing the intensity of fluorescence at the site of each antiviral antibody. The most widely used fluorescent dye in diagnostic virology is fluorescein isothiocyanate (FITC), which emits an intense yellow-green fluorescence, but rhodamine, which emits a deep red fluorescence, is also available. After staining, the specimen is examined under fluorescence microscope with a source of incident UV light [1, 16, 132].

IFA was used for the diagnosis of SARS. The assay showed 100% sensitivity and 100% specificity in the detection of anti-SARS-CoV IgG in human serum samples when compared to RT-PCR [150]. Madhusudana et al. [152] developed IFA for the detection of anti-rabies virus antibodies in human serum and CSF. When compared to the mouse neutralization test, the assay demonstrated a sensitivity of 97.2% and a specificity of 97.9%. IFA was also used for direct detection of HSV antigen in clinical specimens with sensitivity of 84.6% and specificity of 95.7%, compared to the tissue culture method [151]. Moreover, IFA was applied for subtyping of influenza A virus with 100% agreement to RT-PCR [153]. IFA was also used for the detection of EBV [21] and as a confirmatory test for HIV-1 [154]. Concerning DFA, in one study, it showed 60% sensitivity and 96% specificity in the detection of pandemic influenza A (H1N1) pdm09 in children when compared to RT-qPCR [149]. In another study, DFA showed high specificity (99–100%) in comparison to RT-qPCR for the detection of RSV in children [155]. IFA-based commercial test kit (Anti- ZIKV IIFT) is available, developed by Euroimmun AG (Germany), for the detection of ZIKV infection. The assay uses the complete ZIKV particles as antigen. Consequently, cross-reactivities with antibodies against viruses of the flavivirus family can occur. De Ory et al. [156] evaluated the performance of the assay using 126 positive and 102 negative samples. The assay showed 96.8% sensitivity and 72.5% specificity. OXOID Limited (UK) developed DFA-based kit (IMAGEN influenza virus A and B test) for the detection and differentiation of influenza A virus and influenza B virus in human specimens. The assay uses FITC labeled anti-influenza A virus or influenza B virus monoclonal antibodies. The assay has 100% sensitivity and 100% specificity, compared to the cell culture method.

### 3.4. Hemagglutination Inhibition (HI) Assay

**Figure 7:**
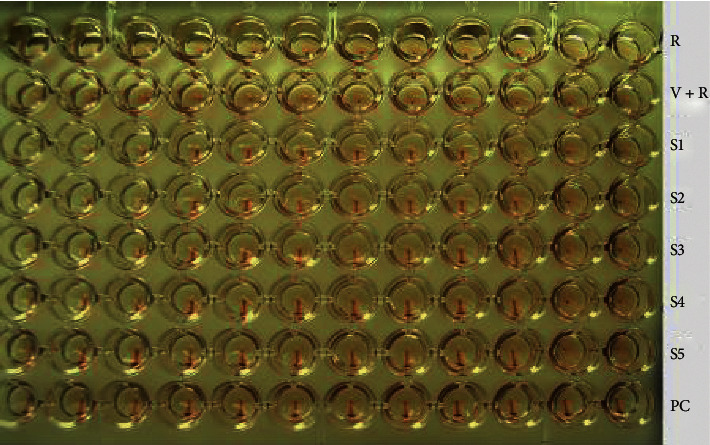
Hemagglutination inhibition assay of avian influenza virus. When the RBCs are alone, they will settle in the bottom of the well. Where specific antibody is absent in the clinical specimen, the viral hemagglutinin is free to agglutinate the RBCs. The HI titer of S1–S5 is 2^10^. Abbreviations: R, RBCs only; V + R, virus and RBCs; PC, positive control; S, sample (unpublished data).

Some viruses such as dengue virus, adenovirus, rubella virus, measles virus, and influenza virus have hemagglutinin antigen on their surfaces that binds and agglutinates RBCs termed hemagglutination (HA). The inhibition of the ability of the viruses to agglutinate RBCs is utilized for the development of HI assay. In the HI assay, serial dilutions of serum sample are prepared in a microtiter plate. Then, a specified amount of viral hemagglutinin is added. Finally, appropriate RBCs are added. The absence of HA indicates a positive reaction. This is judged by tilting the microtiter plate, which allows free RBCs to stream (Figure 7). The dilution rate where complete inhibition of agglutination of RBCs occurred is recorded. The HI titer, therefore, is the reciprocal of the last serum dilution which completely inhibits HA [10, 132, 157]. HI was utilized for a number of applications in diagnostic virology. The assay was used for serosurveillance of influenza A (H1N1) pdm09 virus [158] and measles virus [159]. In one study, HI assay was applied to assess the efficacy of pandemic influenza vaccine [160]. In a validation study using sera from 79 RT-qPCR-confirmed cases and 176 sera from a nonexposed population, HI assay showed high sensitivity (92%) and specificity (91%) for the detection of human infection with 2009 pandemic H1N1 virus [161].

Immunological diagnostic methods are widely employed in routine clinical diagnosis of human viral infections worldwide. The methods have several advantages, such as high sensitivity and specificity, relatively simple to conduct, rapidity, and possibility of testing several specimens simultaneously [10, 138]. However, immunological-based assays do have several limitations. The assays are subject to interferences. Interferences in immunoassays may result from the presence of (a) cross-reactive agents in the sample that carry similar or the same epitopes as the viral antigen of interest, leading to false-positive result [10, 162]; (b) endogenous antibodies, like autoantibodies, heterophilic antibodies, or human anti-animal antibodies in the specimen. Despite the fact that viral antigen is not present in the sample, endogenous antibodies may interact with antiviral antibodies or detection antibodies, leading to false-positive result [162, 163]. The specificity of immunoassay may be affected when they are used in malaria-endemic areas. As it is known, *Plasmodium* induces nonspecific polyclonal B-cell activation that leads to generation of nonspecific antibodies [164]. These broad specific antibodies may react with a variety of antigens, leading to false-positive test. In one study, of 34 samples from PCR confirmed malaria patients, 14 samples were positive or borderline for anti-ZIKV antibodies in commercially available ZIKV ELISA test kit. When these 14 samples were tested using virus neutralization assay, ZIKV infection was not demonstrated in the 11 samples [165]. HI assay is laborious and time-consuming. The interpretation of the assay results between laboratories may be different as no standard reagents are available for the assay [153, 157, 166]. In case of IF assay, prolonged exposure of specimen to UV light leads to fading of fluorescence that could result in false-negative test [167]. Reagents and equipment that are used in some of the immunoassays are expensive [10, 11].

## 4. Status of Diagnostic Methods of Medical Viruses in Ethiopia

Most of viral diseases are endemic to Ethiopia [168]. Serosurveys have demonstrated the high prevalence rate of HBV [169, 170], HCV [170], HIV [171], and HSV-2 [137]. The population is vulnerable to rabies [172] and influenza [173]. Rotaviral diarrhea is the leading cause of morbidity and mortality in children [174]. Recently, like other nations in the globe, public health and economic sectors of Ethiopia are heavily challenged by COVID-19 pandemic.

Immunological methods, mostly commercial ELISA test kits [137, 169] and immunochromatographic test kits [170, 175], are used for the detection of viral infections in most clinical laboratories in the country. IFA technology is available only in Ethiopian Public Health Institute for the detection of rabies virus infection in suspected dogs that bit humans [176]. Conventional RT-PCR is used for the detection of influenza virus in human specimens in National Influenza Laboratory [173]. Recently, the RT-qPCR technique is widely used in several research institutes, universities, and clinical laboratories for the detection of SARS-CoV-2 in clinical samples. In general, few molecular techniques such as conventional PCR and RT-PCR are utilized in research institutes and universities for research purposes. Since most laboratories are budget-restricted and do not have trained laboratory personnel, molecular methods are not used for routine clinical diagnosis of human viral infections in the country. Nationwide use of RT-qPCR technologies for the diagnosis of COVID-19 and the experiences obtained will open the door to introduce molecular techniques for routine laboratory testing of other human viral infections.

## 5. Conclusion

The introduction of nucleic acid-based diagnostic tests into diagnostic virology has made tremendous improvement in the detection of human viral infections. Since nucleic acid-based diagnostic tests are highly sensitive and specific, they play a crucial role in the diagnosis and control of medical viruses. Molecular diagnostic methods diagnose viral infections by detecting viral RNA or DNA. Therefore, these techniques can pick infected individuals before antibody response is mounted against the virus in question. This is especially important in young, elderly, and immunosuppressed patients. However, they are beyond the reach of resource-limited nations due to their high cost, instrumentation complexity, and requirement for technical expertise. Immunoassays also play a significant role in the diagnosis and serosurveillance of viral infections worldwide. Although immunotechniques are easy to perform and inexpensive compared to molecular methods, they are not widely available in low-income countries. Consequently, scientists are working hard to develop inexpensive good quality tests in low-income nations. Moreover, most countries in the developing world are training their citizens abroad and inland at postgraduate level by opening relevant departments and institutes.

## Figures and Tables

**Table 1:** Advantages and limitations of nucleic acid-based amplification techniques.

Detection methods	Advantages	Limitations	References
Conventional PCR	(i) Sensitive and specific	(i) High risk of contamination	[28, 33–35]
	(ii) Widely employed nucleic acid-based detection format	(ii) Prone to inhibitors	
	(iii) Multiplex detection potential	(iii) Time-consuming and labor-intensive	
		(iv) Qualitative	
		(v) Requires thermal cycler and gel documentation apparatus	

Conventional RT-PCR	(i) Sensitive and specific	(i) RNA handling might be difficult	[10, 14, 36–40]
	(ii) Multiplex detection potential	(ii) High risk of contamination	
		(iii) Time-consuming and cumbersome	
		(iv) Relatively expensive	
		(v) Prone to inhibitors	
		(vi) Mutation within PCR primer regions may occur in some RNA viruses which have high mutation rates, leading to reduced sensitivity	

Real-time PCR/RT-qPCR	(i) Highly sensitive and specific	(i) Requires expensive laboratory equipment and fluorescent probe	[33, 34, 39–41]
	(ii) Lower cross-contamination risk due to closed tube operation	(ii) Designing of TaqMan probes requires almost complete information of the target nucleic acid sequence	
	(iii) Rapid and less labor-intensive	(iii) Primer dimer artifact is a problem in case of SYBR green method	
	(iv) Multiplex detection	(iv) Prone to inhibitors	
	(v) Genotyping		
	(vi) Determination of the viral load (quantitative)		

TMA + NASBA	(i) Sensitive and specific	(i) RNA handling might be difficult	[36, 42–45]
	(ii) Simple and rapid (fewer cycles are required)	(ii) Requirement of three enzymes in case of NASBA	
	(iii) Multiplexing potential	(iii) Use of enzymes that are not thermostable	
	(iv) Quantification,	(iv) Nonspecific interactions of the primers may increase as the amplification process occurs at a lower temperature (41°C)	
	(v) Genotyping		
	(vi) Does not require thermal cycler as the reaction takes place isothermally at 41°C		

LAMP/LAMP-RT	(i) Highly sensitive and specific	(i) Requirement of six primers	[35, 46–49]
	(ii) Easy to perform	(ii) High risk of carryover contamination	
	(iii) Does not require expensive thermal cycler	(iii) Limitation for multiplexing	
	(iv) Rapid (results in <1 h)	(iv) Visual detection using naked eye alone is subjective since it depends on observer's perception of color	
	(v) Quantitative		
	(vi) Genotyping		
	(vii) Simple detection systems (using naked eye)		
	(viii) Relatively resistant to inhibitors present in the sample		

## References

[1] Cobo F., Talavera P., Concha Á. (2006). Diagnostic approaches for viruses and prions in stem cell banks. *Virology*.

[2] Owen J. A., Punt J., Stranford S. A., Jones P. P. (2013). *Kuby Immunology, S*.

[3] Suarez D. L., Perdue M. L., Cox N. (1998). Comparisons of highly virulent H5N1 influenza A viruses isolated from humans and chickens from Hong Kong. *Journal of Virology*.

[4] Lipsitch M., Cohen T., Cooper B. (2003). Transmission dynamics and control of severe acute respiratory syndrome. *Science*.

[5] World Health Organization (WHO) (2010). *Pandemic (H1N1) 2009-update 100. Weekly Update*.

[6] Brasil P., Pereira J. P., Moreira M. E. (2016). Zika virus infection in pregnant women in Rio de Janeiro. *New England Journal of Medicine*.

[7] Broadhurst M. J., Brooks T. J. G., Pollock N. R. (2016). Diagnosis of Ebola virus disease: past, present, and future. *Clinical Microbiology Reviews*.

[8] Tan S. K., Sahoo M. K., Milligan S. B., Taylor N., Pinsky B. A. (2017). Stability of Zika virus in urine: specimen processing considerations and implications for the detection of RNA targets in urine. *Journal of Virological Methods*.

[9] Sun K., Chen J., Viboud C. (2020). Early epidemiological analysis of the coronavirus disease 2019 outbreak based on crowdsourced data: a population-level observational study. *The Lancet Digital Health*.

[10] Souf S. (2016). Recent advances in diagnostic testing for viral infections. *Bioscience Horizons*.

[11] Mani R. S., Madhusudana S. N. (2013). Laboratory diagnosis of human rabies: recent advances. *The Scientific World Journal*.

[12] World Health Organization (WHO) (2017). *Global Hepatitis Report 2017*.

[13] World Health Organization (WHO) (2020). Cumulative number of confirmed human cases of avian influenza A (H5N1) reported to WHO. http://www.who.int/influenza/human_animal_interface/H5N1_cumulative_table_archives/en/.

[14] Shen M., Zhou Y., Ye J. (2020). Recent advances and perspectives of nucleic acid detection for coronavirus. *Journal of Pharmaceutical Analysis*.

[15] World Health Organization (WHO) (2020). *Coronavirus Disease 2019 (COVID-19) Situation Report– 156*.

[16] Cella L. N., Blackstock D., Yates M. A., Mulchandani A., Chen W. (2013). Detection of RNA viruses: current technologies and future perspectives. *Critical Reviews in Eukaryotic Gene Expression*.

[17] Landry M., Tang Y., Detrick B., Schmitz J., Hamilton R. (2016). Immunologic and molecular methods for viral diagnosis. *Manual of Molecular and Clinical Laboratory Immunology*.

[18] Acharya T., Kennedy R., Daar A. S., Singer P. A. (2004). Biotechnology to improve health in developing countries: a review. *Memórias Do Instituto Oswaldo Cruz*.

[19] Pankaj K. (2013). Methods for rapid virus identification and quantification. *Materials and Methods*.

[20] Atmar R. L., Kaslow R. A., Stanberry L. R., Le Duc J. W. (2014). Immunological detection and characterization. *Viral Infections of Humans: Epidemiology and Control*.

[21] Zhang S. C., Liu L., Wang R. J. (2014). Detection of Epstein-Barr virus infection subtype in patients with multiple sclerosis by indirect immunofluorescence assay. *Neuroimmunology and Neuroinflammation*.

[22] Chen Y., Chan K. H., Kang Y. (2015). A sensitive and specific antigen detection assay for Middle East respiratory syndrome coronavirus. *Emerging Microbes and Infections*.

[23] Stone C. B., Mahony J. B. (2014). Molecular detection of bacterial and viral pathogens–Where do we go from here?. *Clinical Microbiology: Open Access*.

[24] Canberk S., Longatto-Filho A., Schmitt F. (2016). Molecular diagnosis of infectious diseases using cytological specimens. *Diagnostic Cytopathology*.

[25] Hodinka R. L., Kaiser L. (2013). Point-counterpoint: is the era of viral culture over in the clinical microbiology laboratory?. *Journal of Clinical Microbiology*.

[26] Ender A., Schmitt U. M., Endres W., Luz B., Sugg U. (2004). Screening of blood donations for HIV-1 and HCV RNA by transcription-mediated amplification assay: one year experience. *Transfusion Medicine and Hemotherapy*.

[27] Usawattanakul W., Jittmittraphap A., Endy T. P., Nisalak A., Tapchaisri P., Looareesuwan S. (2003). Rapid detection of dengue viral RNA by nucleic acid sequence-based amplification (NASBA). *Dengue Bulletin*.

[28] Hassan R., White L. R., Stefanoff C. G. (2006). Epstein-Barr Virus (EBV) detection and typing by PCR: a contribution to diagnostic screening of EBV-positive Burkitt’s lymphoma. *Diagnostic Pathology*.

[29] Maignan M., Viglino D., Hablot M. (2019). Diagnostic accuracy of a rapid RT-PCR assay for point-of-care detection of influenza A/B virus at emergency department admission: a prospective evaluation during the 2017/2018 influenza season. *PLoS One*.

[30] Martinez V. M. D. P., Puri V., Oldfield LM., Shabman R. S., Tan G. S., Pickett B. E. (2019). Optimization of qRT-PCR assay for zika virus detection in human serum and urine. *Virus Research*.

[31] Biava M., Colavita F., Marzorati A. (2018). Evaluation of a rapid and sensitive RT-qPCR assay for the detection of Ebola virus. *Journal of Virological Methods*.

[32] Corman V. M., Landt O., Kaiser M. (2020). Detection of 2019 novel coronavirus (2019-nCoV) by real-time RT-PCR. *Eurosurveillance*.

[33] Ramamurthy M., Alexander M., Aaron S. (2011). Comparison of a conventional polymerase chain reaction with real-time polymerase chain reaction for the detection of neurotropic viruses in cerebrospinal fluid samples. *Indian Journal of Medical Microbiology*.

[34] Mackay I. M. (2004). Real-time PCR in the microbiology laboratory. *Clinical Microbiology and Infection*.

[35] Kashir J., Yaqinuddin A. (2020). Loop mediated isothermal amplification (LAMP) assays as a rapid diagnostic for COVID-19. *Medical Hypotheses*.

[36] Lauri A., Mariani P. O. (2009). Potentials and limitations of molecular diagnostic methods in food safety. *Genes and Nutrition*.

[37] Thorburn F., Bennett S., Modha S., Murdoch D., Gunson R., Murcia P. R. (2015). The use of next generation sequencing in the diagnosis and typing of respiratory infections. *Journal of Clinical Virology*.

[38] Nakhaie M., Soleimanjahi H., Mollaie H., Arabzadeh S. (2018). Development of multiplex reverse transcription-polymerase chain reaction for simultaneous detection of influenza A, B and adenoviruses. *Iranian Journal of Pathology*.

[39] Boppana S. B., Ross S. A., Shimamura M. (2011). Saliva polymerase-chain-reaction assay for Cytomegalovirus screening in newborns. *New England Journal of Medicine*.

[40] Bhullar S. S., Chandak N. H., Purohit H. J., Taori G. M., Daginawala H. F., Kashyap R. S. (2014). Determination of viral load by quantitative real-time PCR in herpes simplex encephalitis patients. *Intervirology*.

[41] Singh B. D. (2008). *Biotechnology: Expanding Horizons*.

[42] Fakruddin M. D., Mazumdar R. M., Chowdhury A., Bin Mannan K. S. (2012). Nucleic acid sequence based amplification (NASBA)-prospects and applications. *International Journal of Life science and Pharma Research*.

[43] Sooknanan R., Malek L. T., van Gemen B., Wiedbrauk D. L., Farkas D. H. (1995). Nucleic acid sequence-based amplification. *Methods for Virus Detection*.

[44] Ayele W., Pollakis G., Abebe A. (2004). Development of a nucleic acid sequence-based amplification assay that uses gag-based molecular beacons to distinguish between human immunodeficiency virus type 1 subtype C and C’ infections in Ethiopia. *Journal of Clinical Microbiology*.

[45] Paryan M., Forouzandeh M. M., Kia V., Mohammadi-Yeganeh S., Raz A., Mirab Samiee S. (2013). A simple and rapid method for the detection of HIV-1/HCV in co-infected patients. *Iranian Journal of Biotechnology*.

[46] Hoos J., Peters R. M., Tabatabai J., Grulich-Henn J., Schnitzler P., Pfeil J. (2017). Reverse-transcription loop-mediated isothermal amplification for rapid detection of respiratory syncytial virus directly from nasopharyngeal swabs. *Journal of Virological Methods*.

[47] Bista B. R., Ishwad C., Wadowsky R. M. (2007). Development of a loop-mediated isothermal amplification assay for rapid detection of BK virus. *Journal of Clinical Microbiology*.

[48] Karthik K., Rathore R., Thomas P. (2014). New closed tube loop mediated isothermal amplification assay for prevention of product cross-contamination. *MethodsX*.

[49] Notomi T., Mori Y., Tomita N., Kanda H. (2015). Loop-mediated isothermal amplification (LAMP): principle, features, and future prospects. *Journal of Microbiology*.

[50] Mullis K. B., Faloona F. A. (1987). Specific synthesis of DNA in vitro via a polymerase-catalyzed chain reaction. *Methods in Enzymology*.

[51] Cobo F. (2012). Application of molecular diagnostic techniques for viral testing. *The Open Virology Journal*.

[52] Levine M., Sheu T. G., Gubareva L. V., Mishin V. P. (2011). Detection of hemagglutinin variants of the pandemic influenza A (H1N1) 2009 virus by pyrosequencing. *Journal of Clinical Microbiology*.

[53] Demmler G. J., Buffone G. J., Schimbor C. M., May R. A. (1988). Detection of Cytomegalovirus in urine from newborns by using polymerase chain reaction DNA Amplification. *Journal of Infectious Diseases*.

[54] Myerson D., Lingenfelter P. A., Gleaves C. A., Meyers J. D., Bowden R. A. (1993). Diagnosis of Cytomegalovirus pneumonia by the polymerase chain reaction with archived frozen lung tissue and bronchoalveolar lavage fluid. *American Journal of Clinical Pathology*.

[55] Sundaramurthy R., Dhodapkar R., Kaliaperumal S., Harish B. N. (2018). Investigational approach to adenoviral conjunctivitis: comparison of three diagnostic tests using a Bayesian latent class model. *The Journal of Infection in Developing Countries*.

[56] Gruber F., Falkner F. G., Dorner F., Hämmerle T. (2001). Quantitation of viral DNA by real-time PCR applying duplex amplification, internal standardization, and two-color fluorescence detection. *Applied and Environmental Microbiology*.

[57] Falsey A. R., Formica M. A., Walsh E. E. (2002). Diagnosis of respiratory syncytial virus infection: comparison of reverse transcription-PCR to viral culture and serology in adults with respiratory illness. *Journal of Clinical Microbiology*.

[58] Formenty P., Leroy E. M., Epelboin A. (2006). Detection of Ebola virus in oral fluid specimens during outbreaks of Ebola virus hemorrhagic fever in the Republic of Congo. *Clinical Infectious Diseases*.

[59] Higuchi R., Fockler C., Dollinger G., Watson R. (1993). Kinetic PCR analysis: real-time monitoring of DNA amplification reactions. *Nature Biotechnology*.

[60] Holland P. M., Abramson R. D., Watson R., Gelfand D. H. (1991). Detection of specific polymerase chain reaction product by utilizing the 5′-3′ exonuclease activity of Thermus aquaticus DNA polymerase. *Proceedings of the National Academy of Sciences*.

[61] Espy M. J., Uhl J. R., Sloan L. M. (2006). Real-time PCR in clinical microbiology: applications for routine laboratory testing. *Clinical Microbiology Reviews*.

[62] Gartzonika C., Vrioni G., Priavali E., Pappas G., Levidiotou S. (2012). Utility of Real-time PCR in the diagnosis of primary Epstein-Barr virus infection. *Journal of Medical Microbiology and Diagnosis*.

[63] Dou Y., Li Y., Ma C. (2018). Rapid diagnosis of human adenovirus B, C and E in the respiratory tract using multiplex quantitative polymerase chain reaction. *Molecular Medicine Reports*.

[64] Qiu F. Z., Shen X. X., Zhao M. C. (2018). A triplex quantitative real-time PCR assay for differential detection of human adenovirus serotypes 2, 3 and 7. *Virology Journal*.

[65] Yang J.-H., Lai J.-P., Douglas S. D., Metzger D., Zhu X.-H., Ho W.-Z. (2002). Real-time RT-PCR for quantitation of hepatitis C virus RNA. *Journal of Virological Methods*.

[66] Watzinger F., Suda M., Preuner S. (2004). Real-time quantitative PCR assays for detection and monitoring of pathogenic human viruses in immunosuppressed pediatric patients. *Journal of Clinical Microbiology*.

[67] Bai Z., Liu L., Tu Z. (2008). Real-time PCR for detecting circulating dengue virus in the Guangdong Province of China in 2006. *Journal of Medical Microbiology*.

[68] Gueudin M., Leoz M., Lemée V. (2012). A new real-time quantitative PCR for diagnosis and monitoring of HIV-1 group O infection. *Journal of Clinical Microbiology*.

[69] Behzadi M. A., Ziyaeyan M., Alborzi A. (2016). A diagnostic one-step real-time reverse transcription polymerase chain reaction method for accurate detection of influenza virus type A. *Archives of Medical Science*.

[70] Júnior R. B. M., Carney S., Goldemberg D. (2014). Detection of respiratory viruses by real-time polymerase chain reaction in outpatients with acute respiratory infection. *Memórias do Instituto Oswaldo Cruz*.

[71] Mohammadi-Yeganeh S., Paryan M., Mirab Samiee S., Kia V., Rezvan H. (2012). Molecular beacon probes-base multiplex NASBA Real-time for detection of HIV-1 and HCV. *Iranian Journal of Microbiology*.

[72] Yu A. C.-H., Vatcher G., Yue X. (2012). Nucleic acid-based diagnostics for infectious diseases in public health affairs. *Frontiers of Medicine*.

[73] Mercier‐Delarue S., Vray M., Plantier J. C. (2014). Higher specificity of nucleic acid sequence-based amplification isothermal technology than of real-time PCR for quantification of HIV-1 RNA on dried blood spots. *Journal of Clinical Microbiology*.

[74] Moore C., Telles J.-N., Corden S. (2010). Development and validation of a commercial real-time NASBA assay for the rapid confirmation of influenza A H5N1 virus in clinical samples. *Journal of Virological Methods*.

[75] Swenson P. D., El-Sabaeny A., Thomas-Moricz V. (2016). Evaluation of a transcription mediated amplification assay for detection of herpes simplex virus types 1 and 2 mRNA in clinical specimens. *Journal of Clinical Virology*.

[76] Lee I. S., Choi D. H., Lim J. (2011). Real-time nucleic acid sequence based amplification (Real-time NASBA) for detection of Norovirus. *Journal of Experimental and Biomedical Sciences*.

[77] Moore C., Valappil M., Corden S., Westmoreland D. (2006). Enhanced clinical utility of the NucliSens EasyQ RSV A+B assay for rapid detection of respiratory syncytial virus in clinical samples. *European Journal of Clinical Microbiology and Infectious Diseases*.

[78] Notomi T., Okayama H., Masubuchi H. (2000). Loop-mediated isothermal amplification of DNA. *Nucleic Acids Research*.

[79] Nagamine K., Hase T., Notomi T. (2002). Accelerated reaction by loop-mediated isothermal amplification using loop primers. *Molecular and Cellular Probes*.

[80] Fukuta S., Iida T., Mizukami Y. (2003). Detection of Japanese yam mosaic virus by RT-LAMP. *Archives of Virology*.

[81] Becherer L., Borst N., Bakheit M., Frischmann S., Zengerle R., von Stetten F. (2020). Loop-mediated isothermal amplification (LAMP) - review and classification of methods for sequence specific detection. *Analytical Methods*.

[82] Tomita N., Mori Y., Kanda H., Notomi T. (2008). Loop-mediated isothermal amplification (LAMP) of gene sequences and simple visual detection of products. *Nature Protocols*.

[83] Silva S. J. R. D., Pardee K., Pena L. (2020). Loop-mediated isothermal amplification (LAMP) for the diagnosis of Zika virus: a review. *Viruses*.

[84] Teoh B. T., Sam S. S., Tan K. K. (2013). Detection of dengue viruses using reverse transcription-loop-mediated isothermal amplification. *BMC Infectious Diseases*.

[85] Reddy A. K., Balne P. K., Reddy R. K., Mathai A., Kaur I. (2011). Loop-mediated isothermal amplification assay for the diagnosis of retinitis caused by herpes simplex virus-1. *Clinical Microbiology and Infection*.

[86] Yang J., Fang M.-X., Li J., Lou G.-Q., Lu H.-J., Wu N.-P. (2011). Detection of hepatitis C virus by an improved loop-mediated isothermal amplification assay. *Archives of Virology*.

[87] Shirato K., Yano T., Senba S. (2014). Detection of Middle East respiratory syndrome coronavirus using reverse transcription loop-mediated isothermal amplification (RT-LAMP). *Virology Journal*.

[88] Ziros P. G., Kokkinos P. A., Allard A., Vantarakis A. (2015). Development and evaluation of a loop-mediated isothermal amplification assay for the detection of adenovirus 40 and 41. *Food and Environmental Virology*.

[89] Iwata S., Shibata Y., Kawada J.-i. (2006). Rapid detection of Epstein-Barr virus DNA by loop-mediated isothermal amplification method. *Journal of Clinical Virology*.

[90] Reddy A. K., Balne P. K., Reddy R. K., Mathai A., Kaur I. (2010). Development and evaluation of loop-mediated isothermal amplification assay for rapid and inexpensive detection of Cytomegalovirus DNA in vitreous specimens from suspected cases of viral retinitis. *Journal of Clinical Microbiology*.

[91] Huang W. E., Lim B., Hsu C. C. (2020). RT-LAMP for rapid diagnosis of coronavirus SARS-CoV-2. *Microbial Biotechnology*.

[92] Lu R., Wu X., Wan Z. (2020). Development of a novel reverse transcription loop-mediated isothermal amplification method for rapid detection of SARS-CoV-2. *Virologica Sinica*.

[93] Baek Y. H., Um J., Antigua K. J. C. (2020). Development of a reverse transcription-loop-mediated isothermal amplification as a rapid early-detection method for novel SARS-CoV-2. *Emerging Microbes and Infections*.

[94] Kurosaki Y., Magassouba N., Oloniniyi O. K. (2016). Development and evaluation of reverse transcription-loop-mediated isothermal amplification (RT-LAMP) assay coupled with a portable device for rapid diagnosis of Ebola virus disease in Guinea. *PLoS Neglected Tropical Diseases*.

[95] Zeinoddini M., Monazah A., Saeedinia A. R. (2017). Comparison between RT-PCR, NASBA and RT-LAMP methods for detection of Coxsackievirus B3. *Biomacromolecular Journal*.

[96] Ma X.-j., Shu Y.-l., Nie K. (2010). Visual detection of pandemic influenza A H1N1 Virus 2009 by reverse-transcription loop-mediated isothermal amplification with hydroxynaphthol blue dye. *Journal of Virological Methods*.

[97] Dinh D. T., Le M. T. Q., Vuong C. D., Hasebe F., Morita K. (2011). An updated loop-mediated isothermal amplification method for rapid diagnosis of H5N1 avian influenza viruses. *Tropical Medicine and Health*.

[98] Kargar M., Askari A., Doosti A., Ghorbani-Dalini S. (2012). Loop-mediated isothermal amplification assay for rapid detection of hepatitis C virus. *Indian Journal of Virology*.

[99] Rudolph D. L., Sullivan V., Owen S. M., Curtis K. A. (2015). Detection of acute HIV-1 infection by RT-LAMP. *PLoS One*.

[100] Sabalza M., Yasmin R., Barber C. A. (2018). Detection of Zika virus using reverse-transcription LAMP coupled with reverse dot blot analysis in saliva. *PLoS One*.

[101] Herrera-Rodriguez S. E., Elizondo-Quiroga D., Alvarez-Maya I. (2013). Infectious diseases detection by microarray: an overview of clinical relevant infections. *Journal of Biomedical Science and Engineering*.

[102] Chiu C. Y., Urisman A., Greenhow T. L. (2008). Utility of DNA microarrays for detection of viruses in acute respiratory tract infections in children. *The Journal of Pediatrics*.

[103] Müller R., Ditzen A., Hille K. (2009). Detection of herpesvirus and adenovirus co-infections with diagnostic DNA-microarrays. *Journal of Virological Methods*.

[104] Boriskin Y. S., Rice P. S., Stabler R. A. (2004). DNA microarrays for virus detection in cases of central nervous system infection. *Journal of Clinical Microbiology*.

[105] Martínez M. A., Soto-del Río M. d. l. D., Gutiérrez R. M. (2015). DNA microarray for detection of gastrointestinal viruses. *Journal of Clinical Microbiology*.

[106] Khan M. J., Trabuco A. C., Alfonso H. L. (2016). DNA microarray platform for detection and surveillance of viruses transmitted by small mammals and arthropods. *PLoS Neglected Tropical Diseases*.

[107] Korimbocus J., Scaramozzino N., Lacroix B., Crance J. M., Garin D., Vernet G. (2005). DNA probe array for the simultaneous identification of herpesviruses, enteroviruses, and flaviviruses. *Journal of Clinical Microbiology*.

[108] Granade T. C., Kodani M., Wells S. K. (2018). Characterization of real-time microarrays for simultaneous detection of HIV-1, HIV-2, and hepatitis viruses. *Journal of Virological Methods*.

[109] Díaz-Badillo A., de Lourdes Muñoz M., Perez-Ramirez G. (2014). A DNA microarray-based assay to detect dual infection with two dengue virus serotypes. *Sensors*.

[110] Martín V., Perales C., Fernàndez-Algar M. (2016). An efficient microarray-based genotyping platform for the identification of drug-resistance mutations in majority and minority subpopulations of HIV-1 Quasispecies. *PLoS One*.

[111] Masimba P., Gare J., Klimkait T., Tanner M., Felger I. (2014). Development of a simple microarray for genotyping HIV-1 drug resistance mutations in the reverse transcriptase gene in rural Tanzania. *Tropical Medicine and International Health*.

[112] Hua W., Zhang G., Guo S., Li W., Sun L., Xiang G. (2015). Microarray-based genotyping and detection of drug-resistant HBV mutations from 620 Chinese patients with chronic HBV infection. *The Brazilian Journal of Infectious Diseases*.

[113] Guo X., Geng P., Wang Q., Cao B., Liu B. (2014). Development of a single nucleotide polymorphism DNA microarray for the detection and genotyping of the SARS coronavirus. *Journal of Microbiology and Biotechnology*.

[114] Dankbar D. M., Dawson E. D., Mehlmann M. (2007). Diagnostic microarray for influenza B viruses. *Analytical Chemistry*.

[115] Wang D., Urisman A., Liu Y. T. (2003). Viral discovery and sequence recovery using DNA microarrays. *PLoS Biology*.

[116] Bexfield N., Kellam P. (2011). Metagenomics and the molecular identification of novel viruses. *The Veterinary Journal*.

[117] Bumgarner R. (2013). DNA microarrays: types, applications and their future. *Current Protocols in Molecular Biology*.

[118] Lefterova M. I., Suarez C. J., Banaei N., Pinsky B. A. (2015). Next-generation sequencing for infectious disease diagnosis and management. *The Journal of Molecular Diagnostics*.

[119] Vemula S., Zhao J., Liu J., Wang X., Biswas S., Hewlett I. (2016). Current approaches for diagnosis of influenza virus infections in humans. *Viruses*.

[120] Deurenberg R. H., Bathoorn E., Chlebowicz M. A. (2017). Application of next generation sequencing in clinical microbiology and infection prevention. *Journal of Biotechnology*.

[121] Laver T., Harrison J., O’Neill P. A. (2015). Assessing the performance of the Oxford nanopore technologies MinION. *Biomolecular Detection and Quantification*.

[122] Jain M., Koren S., Miga K. H. (2018). Nanopore sequencing and assembly of a human genome with ultra-long reads. *Nature Biotechnology*.

[123] Ji W., Hua K. Y., Yong Z. (2017). Rapid and accurate sequencing of enterovirus genomes using MinION nanopore sequencer. *Biomedical and Environmental Sciences*.

[124] Imai K., Tamura K., Tanigaki T. (2018). Whole genome sequencing of influenza A and B viruses with the MinION sequencer in the clinical setting: a pilot study. *Frontiers in Microbiology*.

[125] Quick J., Grubaugh N. D., Pullan S. T. (2017). Multiplex PCR method for MinION and Illumina sequencing of Zika and other virus genomes directly from clinical samples. *Nature Protocols*.

[126] Kustin T., Ling G., Sharabi S. (2019). A method to identify respiratory virus infections in clinical samples using next-generation sequencing. *Scientific Reports*.

[127] Baillie G. J., Galiano M., Agapow P.-M. (2012). Evolutionary dynamics of local pandemic H1N1/2009 influenza virus lineages revealed by whole-genome analysis. *Journal of Virology*.

[128] Dessilly G., Goeminne L., Vandenbroucke A.-t, Dufrasne F. E., Martin A., Kabamba-Mukabi B. (2018). First evaluation of the Next-Generation Sequencing platform for the detection of HIV-1 drug resistance mutations in Belgium. *PLoS One*.

[129] Towner J. S., Sealy T. K., Khristova M. L. (2008). Newly discovered Ebola virus associated with hemorrhagic fever outbreak in Uganda. *PLoS Pathogens*.

[130] Lin Z., Farooqui A., Li G. (2014). Next-generation sequencing and bioinformatic approaches to detect and analyze influenza virus in ferrets. *The Journal of Infection in Developing Countries*.

[131] Jerome H., Taylor C., Sreenu V. B. (2019). Metagenomic next-generation sequencing aids the diagnosis of viral infections in febrile returning travellers. *Journal of Infection*.

[132] Kindt T. J., Goldsby R. A., Osborne B. A. (2007). *Kuby Immunology*.

[133] Huang Y., Zhu Y., Yang M., Zhang Z., Song D., Yuan Z. (2014). Nucleoprotein-based indirect enzyme-linked immunosorbent assay (indirect ELISA) for detecting antibodies specific to Ebola virus and Marbug virus. *Virologica Sinica*.

[134] Adams E. R., Ainsworth M., Anand R. (2020). Evaluation of antibody testing for SARS-CoV-2 using ELISA and lateral flow immunoassays. *MedRxiv*.

[135] Colavita F., Brogi A., Lapa D. (2020). Evaluation of ELISA tests for the qualitative determination of IgG, IgM and IgA to SARS-CoV-2. *MedRxiv*.

[136] Lai S.-C., Huang Y.-Y., Shu P.-Y. (2019). Development of an enzyme-linked immunosorbent assay for rapid detection of dengue virus (DENV) NS1 and differentiation of DENV serotypes during early infection. *Journal of Clinical Microbiology*.

[137] Kebede Y., Dorigo-Zetsma W., Mengistu Y. (2004). Transmission of Herpes simplex virus type 2 among factory workers in Ethiopia. *The Journal of Infectious Diseases*.

[138] Lau S. K. P., Woo P. C. Y., Wong B. H. L. (2004). Detection of severe acute respiratory syndrome (SARS) coronavirus nucleocapsid protein in SARS patients by enzyme-linked immunosorbent assay. *Journal of Clinical Microbiology*.

[139] Amado L. A., Villar L. M., de Paula V. S., Almeida A. J. d., Gaspar A. M. C. (2006). Detection of hepatitis A, B, and C virus-specific antibodies using oral fluid for epidemiological studies. *Memórias Do Instituto Oswaldo Cruz*.

[140] Ohnishi K., Takahashi Y., Kono N. (2012). Newly established monoclonal antibodies for immunological detection of H5N1 influenza virus. *Japanese Journal of Infectious Diseases*.

[141] Huzly D., Hanselmann I., Schmidt-Chanasit J., Panning M. (2016). High specificity of a novel Zika virus ELISA in European patients after exposure to different flaviviruses. *Eurosurveillance*.

[142] Buttò S., Suligoi B., Fanales-Belasio E., Raimondo M. (2010). Laboratory diagnostics for HIV infection. *Annali dell’Istituto Superiore di Sanità*.

[143] He Q., Chong K. H., Chng H. H. (2004). Development of a Western blot assay for detection of antibodies against coronavirus causing severe acute respiratory syndrome. *Clinical Diagnostic Laboratory Immunology*.

[144] Yang Z., Lee J., Ahn H.-J., Chong C.-K., Dias R. F., Nam H.-W. (2016). Western blot detection of human anti-Chikungunya virus antibody with recombinant envelope 2 protein. *The Korean Journal of Parasitology*.

[145] Huang J., Wang M., Huang C. (2018). Western blot-based logistic regression model for the identification of recent HIV-1 infection: a promising HIV-1 surveillance approach for resource-limited regions. *BioMed Research International*.

[146] Yeh C. T., Han C. M., Lo S. Y. (1994). Early detection of anti-HCc antibody in acute hepatitis C virus (HCV) by western blot (immunoblot) using a recombinant HCV core protein fragment. *Journal of Clinical Microbiology*.

[147] Jackson J. B., Parsons J. S., Nichols L. S., Knoble N., Kennedy S., Piwowar E. M. (1997). Detection of human immunodeficiency virus type 1 (HIV-1) antibody by western blotting and HIV-1 DNA by PCR in patients with AIDS. *Journal of Clinical Microbiology*.

[148] Sudha T., Lakshmi V., Teja V. D. (2006). Western blot profile in HIV infection. *Indian Journal of Dermatology, Venereology and Leprology*.

[149] Nitsch-Osuch A., Woźniak-Kosek A., Brydak L. (2012). Accuracy of rapid influenza diagnostic test and immunofluorescence assay compared to real time RT-PCR in children with influenza A(H1N1)pdm09 infection. *Postępy Higieny I Medycyny Doświadczalnej*.

[150] Chan P. K. S., Ng K.-C., Chan R. C. W. (2004). Immunofluorescence assay for serologic diagnosis of SARS. *Emerging Infectious Diseases*.

[151] Pouletty P., Chomel J. J., Thouvenot D., Catalan F., Rabillon V., Kadouche J. (1987). Detection of herpes simplex virus in direct specimens by immunofluorescence assay using a monoclonal antibody. *Journal of Clinical Microbiology*.

[152] Madhusudana S. N., Shamsundar R., Saraswati S. (2001). Comparative evaluation of a simple indirect immunofluorescence test and mouse neutralization test for assaying rabies antibodies. *Indian Journal of Pathology and Microbiology*.

[153] Johnson J., Higgins A., Navarro A. (2012). Subtyping influenza A virus with monoclonal antibodies and an indirect immunofluorescence assay. *Journal of Clinical Microbiology*.

[154] Kiptoo M. K., Mpoke S. S., Ng’ang’a Z. W. (2004). New indirect immunofluorescence assay as a confirmatory test for human immunodeficiency virus type 1. *East African Medical Journal*.

[155] Shafik C. F., Mohareb E. W., Youssef F. G. (2011). Comparison of direct fluorescence assay and real-time RT-PCR as diagnostics for respiratory syncytial virus in young children. *Journal of Tropical Medicine*.

[156] De Ory F., Sánchez-Seco M., Vázquez A. (2018). Comparative evaluation of indirect immunofluorescence and NS-1-based ELISA to determine Zika virus-specific IgM. *Viruses*.

[157] Mather S., Scott S., Temperton N., Wright E., King B., Daly J. (2013). Current progress with serological assays for exotic emerging/re-emerging viruses. *Future Virology*.

[158] Alladi C. S. H., Jagadesh A., Prabhu S. G., Arunkumar G. (2019). Hemagglutination inhibition antibody response following influenza A(H1N1)pdm09 virus natural infection: a cross-sectional study from thirthahalli, Karnataka, India. *Viral Immunology*.

[159] Numazaki K. (2015). Study on assays for the detection of serum antibodies to measles from children and its standardization. *International Journal of Pediatrics and Neonatal Care*.

[160] Noah D. L., Hill H., Hines D., White E. L., Wolff M. C. (2009). Qualification of the hemagglutination inhibition assay in support of pandemic influenza vaccine licensure. *Clinical and Vaccine Immunology*.

[161] Veguilla V., Hancock K., Schiffer J. (2011). Sensitivity and specificity of serologic assays for detection of human infection with 2009 pandemic H1N1 virus in U.S. populations. *Journal of Clinical Microbiology*.

[162] Schiettecatte J., Anckaert E., Smitz J., Chiu N. H. L., Christopoulos T. K. (2012). Interferences in immunoassays. *Advances in Immunoassay Technology*.

[163] Emerson J. F., Lai K. K. Y. (2013). Endogenous antibody interferences in immunoassays. *Laboratory Medicine*.

[164] Scholzen A., Sauerwein R. W. (2013). How malaria modulates memory: activation and dysregulation of B cells in *Plasmodium* infection. *Trends in Parasitology*.

[165] Van Esbroeck M., Meersman K., Michiels J., Ariën K. K., Van den Bossche D. (2016). Letter to the editor: specificity of Zika virus ELISA: interference with malaria. *Eurosurveillance*.

[166] Upadhyay C., Ammayappan A., Vakharia V. N. (2009). Detection of NP, N3 and N7 antibodies to avian influenza virus by indirect ELISA using yeast-expressed antigens. *Virology Journal*.

[167] Ogilvie M. M., Burnett D., Crocker J. (2005). Microscopy in virology: application and sample preparation. *The Science of Laboratory Diagnosis*.

[168] Aseffa A. (1993). Viral diseases in Ethiopia: a review. *East African Medical Journal*.

[169] Abebe A., Nokes D. J., Dejene A., Enquselassie F., Messele T., Cutts F. T. (2003). Seroepidemiology of hepatitis B virus in Addis Ababa, Ethiopia: transmission patterns and vaccine control. *Epidemiology and Infection*.

[170] Ayele A. G., Gebre-Selassie S. (2013). Prevalence and risk factors of hepatitis B and hepatitis C virus infections among patients with chronic liver diseases in public hospitals in Addis Ababa, Ethiopia. *ISRN Tropical Medicine*.

[171] Kenyon C. R., Tsoumanis A., Schwartz I. S. (2015). HIV prevalence correlates with high-risk sexual behavior in Ethiopia’s regions. *PLoS One*.

[172] Deressa A., Pal M., Mamo H., Haile A., Dasgupta R. (2015). Rabies: a major fatal viral disease of humans and animals in Ethiopia. *Journal of Natural History*.

[173] Ayele W., Demissie G., Kassa W. (2012). Challenges of establishing routine influenza sentinel surveillance in Ethiopia, 2008-2010. *Journal of Infectious Diseases*.

[174] Ramos J. M., Alegria I., Tessema D. (2015). Epidemiology of Rotavirus diarrhea among children aged less than 5 years in rural southern Ethiopia. *The Southeast Asian Journal of Tropical Medicine and Public Health*.

[175] Belete W., Deressa T., Feleke A. (2019). Evaluation of diagnostic performance of non-invasive HIV self-testing kit using oral fluid in Addis Ababa, Ethiopia: a facility-based cross-sectional study. *PLoS One*.

[176] Ali A., Mengistu F., Hussen K. (2010). Overview of rabies in and around Addis Ababa, in animals examined in EHNRI zoonoses laboratory between, 2003 and 2009. *Ethiopian Veterinary Journal*.

